# Relationship between the Composition of Lipids in Forages and the Concentration of Conjugated Linoleic Acid in Cow’s Milk: A Review

**DOI:** 10.3390/ani12131621

**Published:** 2022-06-24

**Authors:** Isabel Cristina Acosta Balcazar, Lorenzo Danilo Granados Rivera, Jaime Salinas Chavira, Benigno Estrada Drouaillet, Miguel Ruiz Albarrán, Yuridia Bautista Martínez

**Affiliations:** 1 Facultad de Medicina Veterinaria y Zootecnia, Universidad Autónoma de Tamaulipas, Ciudad Victoria 87000, Tamaulipas, Mexico; a2213058001@alumnos.uat.edu.mx (I.C.A.B.); jsalinas@docentes.uat.edu.mx (J.S.C.); miguel.ruiz@docentes.uat.edu.mx (M.R.A.); 2 Instituto Nacional de Investigaciones Forestales, Agrícolas y Pecuarias, General Terán 67400, Nuevo León, Mexico; 3 Facultad de Ingeniería y Ciencias, Universidad Autónoma de Tamaulipas, Ciudad Victoria 87000, Tamaulipas, Mexico; benestrada@docentes.uat.edu.mx

**Keywords:** biohydrogenation, conjugated linoleic acid, fatty acids, forage, grasses

## Abstract

**Simple Summary:**

Conjugated linoleic acid (CLA) has been shown to have protective effects against various common diseases, such as obesity and cancer, improving human health. For several years, efforts have been made to increase CLA levels in milk by including sources of fats and oilseeds in the diets of lactating cows, causing a decrease in the amount of fat in the milk itself and a decrease in the yield of the products derived from it. A “safe” and economical way to increase CLA content without affecting fat content, is through grazing feeding since the content of CLA precursors (linoleic and α-linolenic acids) are present in greater quantity in pastures compared to feeding only balanced diets. The content of these precursors will depend on factors, such as age and nitrogen fertilization, since the high availability of nitrogen stimulates the synthesis of metabolic components, such as leaf protein. The bibliographic review shows how the inclusion of different forages and the agronomic management of pastures promote the improvement of CLA levels in milk, giving “an added value”.

**Abstract:**

Conjugated linoleic acid (CLA), has been shown to have protective effects against various diseases, such as obesity, arteriosclerosis, diabetes, chronic inflammatory diseases, and cancer. This fatty acid in ruminants results from two processes, biohydrogenation, which takes place in the rumen, and de novo synthesis, carried out in the mammary gland, and it has linoleic and α-linolenic acids as its precursors. The amounts of precursors in the diets of animals are related to the amounts of CLA in milk. In the literature review, it was found that the milk of cows fed fresh forage has a higher amount of CLA because they have a higher amount of linoleic acid and α-linolenic acid compared to other foods used in the diets of cows. The amount of CLA precursors in pastures can be increased through agronomic practices, such as nitrogen fertilization, and regrowth age. It is also a technique used to increase the amount of CLA in milk to obtain a greater benefit regarding its nutritional value.

## 1. Introduction

Milk is a basic food in human nutrition [1] and provides essential nutrients with high bioavailability and does not represent a health risk since the industrial processes to which it is subjected allow it to be harmless [2]. Approximately 98% of milk fat is triglycerides of which 70% are saturated fatty acids (FA) [3]. This has been related to an increase in cholesterol concentration, causing diseases, such as type-2 diabetes, cancer, heart disease, and obesity [4]. In addition, milk contains CLA, a polyunsaturated fatty acid that is produced in the rumen by biohydrogenation [5] and has been shown to have protective effects for some diseases [6]; for this reason, strategies have been sought to increase CLA in milk and dairy products.

The fat synthesis will depend on the effect of the type of feed given to animals (grass, green forage, silage, oilseeds, supplements with fats, or the use of vitamin–mineral supplements; [7]. For example, oils and seeds with a high content of linoleic acid increase the content of linoleic acid in milk fat, while the effect on the content of α-linolenic acid is negative in the case of oils and slightly positive with seeds [8].

In the specific case of fresh pastures, there is evidence that they increase the concentration of CLA in ruminant milk [9]; this is because grasses have high concentrations of linoleic and α-linolenic acids [10]. These FAs can be increased in pastures through management techniques that promote rapid vegetative growth [11]. Among these are the management of regrowth age and nitrogen fertilization [12]. For example, with the consumption of young forages, an increase is expected in the concentration of CLA in ruminant milk compared to the consumption of mature forages [13].

To this regard, León et al. [10] proposed that high availability of nitrogen for plants stimulates the production of dry matter, increases the number of leaves, and stimulates the synthesis of metabolic components such as chlorophyll. Based on these antecedents, the present literature review has the objective of systematizing and synthesizing information regarding the effect that FAs of forages have on the concentration of CLA in cow’s milk to trace research lines that seek to increase FA in pastures that are precursors of CLA in cow’s milk.

## 2. World Overview of Milk Production and Consumption

The world milk production is divided into two large groups, one made up of European countries and the United States, which have numerous subsidy programs; the other country groups with low production costs due to the agroclimatic conditions for efficient production, such as Australia, New Zealand, Argentina, and Uruguay [14]. In this regard, in 2018, the European Union had a 30.6% share, standing out as the main milk-producing region, while Canada was one of the countries with the lowest production [15].

Around 6 billion people in the world consume milk and dairy products [16]. India was the largest consumer with 26.7%, followed by the European Union with 26.2% [15].

## 3. Importance of Milk in Human Diet

Cow’s milk is a basic food in the human diet and has been part of our diet for the last 10,000 years [1]. Throughout life, animal milk (cow, goat, sheep, among others) and its byproducts are included as part of a correct diet since they provide essential nutrients of high bioavailability, are accessible, pleasant to the senses, and enjoy great acceptance in most cultures [2]. However, both milk and its byproducts have a high content of saturated FAs, which can represent 65% of the total FAs [6], and these have been related to an increase in the concentration of cholesterol, causing diseases such as type-2 diabetes, cancer, heart disease, and obesity [4].

In contrast, the International Agency for Research on Cancer has a classification of substances that are suspected of causing cancer and divides them into five categories based on the degree of carcinogenicity. In this regard, milk does not appear in any category [17].

Dairy consumption reduces the incidence of cardiovascular diseases despite its high content of saturated FAs [18]. Likewise, an increase in dairy intake was associated with 0.65% less body fat and a 13% lower risk of being overweight or obese [19]. These effects are attributed to the conjugated linoleic acid (CLA) present in milk, which also works as an antitumor and antiarteriosclerotic agent [20].

## 4. Fat Synthesis

The nutritional relevance of milk is due to the lipid and protein fractions. The lipid fraction is made of saturated, monounsaturated, and polyunsaturated FAs, while the protein fraction includes caseins, whey proteins, and fat globule membrane proteins [3]. Lipids are one of the most important compounds in milk since they have unique characteristics of flavor, nutritional content, and physical properties. Furthermore, they are a good source of energy and an excellent means of transport for fat-soluble vitamins A, D, E, and K [2]. The fat content in milk varies from 3 to 4% and this fraction is composed of 98% triglycerides, on average, which are synthesized in the mammary gland, and of these, 70% correspond to saturated FAs, 26% to monounsaturated, and 4% to polyunsaturated FAs [3].

Milk fat is synthesized directly in the mammary gland from acetate, β-hydroxybutyrate, fatty acids, and, to a lesser extent, glucose [21]. The FAs come from the transformations that food undergoes in the rumen. One of the initial transformations is carried out through lipolysis where esterified FAs are released in triglycerides, glycolipids, and phospholipids due to the effect of hydrolysis. Biohydrogenation is next, where a reduction of the existing double bonds in the released FAs is carried out [22]. FAs from C4 to C10 are synthesized de novo in the mammary gland and use acetic and butyric FAs as precursors, while FAs from C12 to C16 are both synthesized in the mammary gland and transported in the blood through a non-covalent bond with serum albumin; C16 synthesis can also occur in the intestines or in fatty tissue [3].

The de novo synthesis in the mammary gland requires that acetate (from ruminal fermentation) be activated to acetyl-CoA by the acetyl-CoA synthetase enzyme and subsequently be carboxylated by the acetyl-CoA carboxylase enzyme to form malonyl-CoA [22]. After the carbon chain elongation occurs, the chain is built from malonyl-CoA acetyl-CoA in the presence of NADPH; the process involves four stages: condensation, reduction, dehydration, and re-reduction. In each phase, two carbon atoms are attached to the chain [7]. Once triglycerides are made, they begin to join and form microdroplets, which are released as small fat globules (>0.5 μm) or larger globules (0.5 to 15 μm). When the fat globules are located near the apical membrane of the lactocyte, they are surrounded by the cytoplasmic membrane of the secretory cell and are released into the alveolar lumen [21].

### 4.1. Ruminal Biohydrogenation

**Figure 1:**
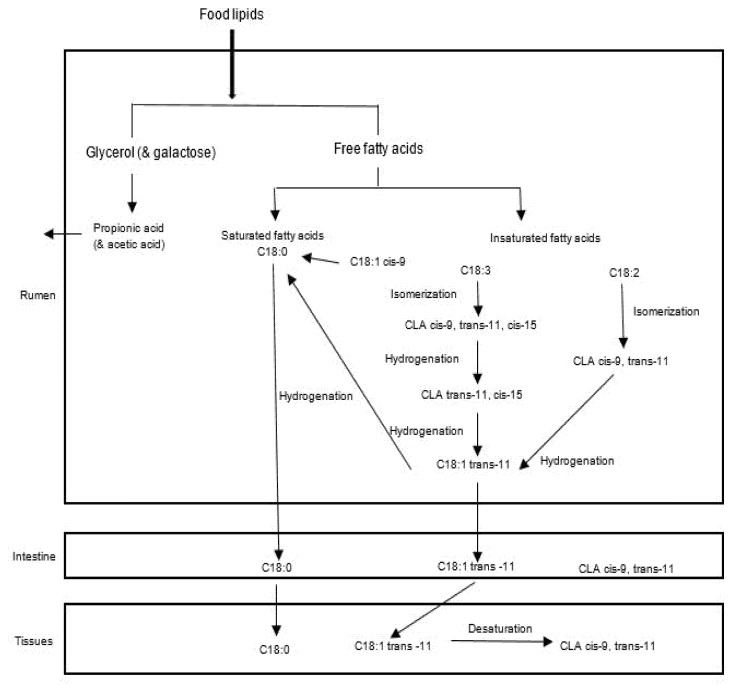
Scheme of ruminal digestion of lipids [22].

After the lipolysis process, the released polyunsaturated and monounsaturated FAs are transformed through hydrogenation [23]. Biohydrogenation is a process that takes place in the rumen, consisting of the saturation (addition of hydrogen) of the double bonds present in FAs [22] and involves several biochemical steps, with speeds, intermediate, and characteristic bacterial species [24]. This activity is mainly associated with bacteria bound to feed particles; the free unsaturated FAs are absorbed on feed particle surfaces and hydrogenated [25], while the saturated FAs are not modified in the rumen. Biohydrogenation is a defense mechanism that rumen microorganisms have to combat the toxicity that polyunsaturated FAs represent to them [20]. The main substrates of this process are linoleic and α-linolenic acids, and it leads to the formation of stearic acid, Figure 1 [22].

### 4.2. Synthesis of Conjugated Linoleic Acid (CLA)

The CLA is a group of geometric and positional isomers of linoleic acid [C18H32O2; cis-9, cis-12; 6] of which the cis-9 trans-11 isomer is the one found in the highest percentage (75 to 90% of CLA [26]). It has two origins: (1) from biohydrogenation, which occurs in the rumen, and (2) from endogenous synthesis in the mammary gland [5].

Biohydrogenation is the main process of CLA synthesis and begins with the isomerization of the cis-12 to trans-11 bond by the action of the linoleate isomerase enzyme, resulting in variable proportions of isomers of CLA [cis-9 trans-11, trans-9 cis-11, trans-10 cis-12, among others; 4]. Afterward, the hydrogenation of the cis-9 bond is carried out to form vaccenic acid, although in the process of α-linolenic acid cis-15, it is also hydrogenated [22]. In the last step, the transformation of vaccenic acid to stearic acid takes place.

The reduction of vaccenic acid seems to be the determining step in the biohydrogenation of linoleic and α-linolenic acids, and therefore, this intermediate could accumulate in the rumen, thus increasing its availability to be absorbed [24].

Recent studies have reported that there is an alternative pathway for the formation of CLA in non-ruminant animals, and it is through the FADS3 gene. This gene prevents the desaturation of trans-vaccenic acid and causes the production of the trans-11, cis-13 isomer of CLA [27].

**Figure 2:**
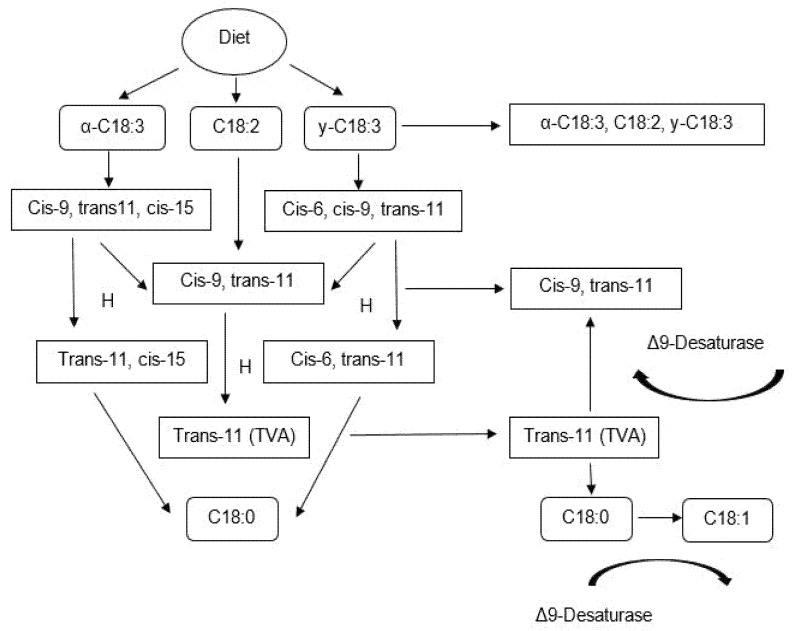
Synthesis of conjugated linoleic acid in ruminants [26].

In terms of the de novo synthesis, the monounsaturated FA formed in the rumen can subsequently be transformed into CLA in the mammary gland from the effect of desaturation [28]. This process requires that acetate (from ruminal fermentation) be activated to acetyl-CoA by the acetyl-CoA synthetase enzyme and subsequently be carboxylated by the acetyl-CoA carboxylase enzyme to form malonyl-CoA, Figure 2 [22].

### 4.3. Factors That Affect Milk Composition

The production, chemical composition, and the FA profile of milk are affected by intrinsic factors related to the animal itself, such as the genotype, period, number of lactations, age, and diseases [29]. For example, fat and protein are genetic components with high heritability [30]. On the other hand, the composition of the fatty acid profile of milk is modified by the process of de novo synthesis, which occurs in the mammary gland of the cow since it has been found that this synthesis has a positive relationship with the formation of medium chain fatty acids and a negative relationship with the formation of long-chain fatty acids [31]. Variations in energy metabolism, such as a negative energy balance after calving and in early lactation, also modify milk fat concentration and lipid profile due to the uptake of non-esterified fatty acids by the mammary gland caused by a high mobilization of reserve fat; however, the percentage of fat will progressively decrease due to the effect of dilution and a decrease in the mobilization of reserve fat [32].

## 5. Influence of Diet on the Profile of Fatty Acids in Milk

Feeding is the most important component in milk production systems due to the high energy demand of animals to maintain production [33]. These systems depend to a great extent on forage resources since around 90% of the nutrients required by animals are derived from pastures [34]. Likewise, the feed provides, directly or indirectly, the nutrients that are precursors of the solid components of milk, such as fat [35].

The FA profile (including CLA content) is affected by feed type (grass, green forage, silage), plant species, supplementation with oils or oilseeds, and the use of vitamin–mineral supplements [7]. Regarding this, Roca et al. [29] mentioned that in oilseeds (soybean, sunflower, rapeseed, and cotton) the main component is linoleic acid, while α-linolenic acid is found in flaxseed and fresh forage; these being the main FA precursors of CLA synthesis.

However, the quality and availability of forage in the grazing grounds are not constant throughout the year. In the lower temperature months, there is less grass production, and in some places, in summer its nutritional quality decreases [36]. Therefore, it is necessary to use feeding techniques that help contribute the nutrients that the animals require, for example, the use of forage silage with grains, such as sorghum and corn and forage grasses. Well-managed forages are a complete feed for cows and allow good milk production [37].

### 5.1. Synthesis of Fatty Acids in Plants and Seeds

The lipid fraction in forages is made up of 33% simple lipids (diglycerides), 50% galactolipids (mono and digalactoglycerides), and 17% phospholipids [10]. In both plants and seeds, the de novo synthesis of FAs is carried out in the plastids by the action of the Acetyl-CoA carboxylase enzyme and a multienzyme complex called fatty acid synthase [38].

The Acetyl-CoA carboxylase enzyme synthesizes malonyl-CoA through carboxylation of an Acetyl-CoA molecule. Then, another enzyme, called β-ketoacyl synthase III (KASIII), condenses an Acetyl-CoA molecule to the malonyl group, undergoing decarboxylation, forming a 4-carbons-long intermediate. The ketoacyl-ACP (Acill Carrier Protein) formed in each cycle undergoes a reduction (generating the reaction intermediate hydroxyacyl-ACP), dehydration (generating transenoyl-ACP), and again another reduction (generating acyl-ACP) [39]. The fundamental sequence of reactions through which long FA chains are built consists of four stages: condensation, reduction of the carbonyl group, dehydration, and reduction of the double bond [38].

These reactions are carried out by the ketoacyl-ACP reductase, hydroxyacyl-ACP dehydratase, and enoyl-ACP reductase enzymes, respectively. In this way, in each cycle of reactions, the carbon chain is lengthened by two carbon atoms [39].

Once synthesized, FAs are transported to the endoplasmic reticulum for elongation. Two pathways have been suggested for such transport: (1) through spontaneous desorption, diffusion, and absorption since in many plant species and cell types, the endoplasmic reticulum has been found close to the chloroplasts, which may facilitate the transfer of FA to the reticulum, and (2) the transport of lipids to the endoplasmic reticulum could happen due to the Acyl-CoA “binding protein” (ACBPs; [40]).

### 5.2. Effect of Incorporating Seeds and Vegetable Oils in the Diets on CLA Content in Milk

Vegetable sources of fat (oil seeds and vegetable oils) are the most indicated to modify the FA profile of milk through the diet [8]. Moreover, oilseeds are used to increase energy intake and the efficiency of milk fat synthesis [41]. The effect of supplementation with oilseeds on the proportion of fat in milk depends on the species, its form or treatment (extruded, cold meal, whole unprocessed), and the interaction with the type of base diet of grass silage, hay, or pasture [42] (Table 1).

When unprocessed seeds are included, there are small increases in CLA in milk since there is little interaction of polyunsaturated FAs at the ruminal level for the production of the main precursor of CLA, that is, vaccenic acid [42]. The extruded seeds reduce the percentage of milk fat due to a greater exposure of the oil to ruminal microorganisms [52]. With the application of heat, the seeds can denature the protein matrix that surrounds the fat droplets and thus protect it from ruminal biohydrogenation, allowing the increase of polyunsaturated FAs in milk [51]. With the increase in unsaturated FAs in the diet, the production of volatile FAs in the rumen decreases; thus, there is less acetate to carry out the de novo synthesis of short- and medium-saturated FA chains in the mammary cells [8].

Vegetable oils with high contents of linoleic and α-linolenic acids (from soybean, cotton, sunflower, flax, safflower, and rapeseed) are the most suitable for increasing CLA in milk [42]. To increase CLA in the rumen, the oils must be available for the microorganisms responsible for ruminal biohydrogenation, as is the case with extruded oils [53]. The effects produced by vegetable oils on milk fat are: (1) decrease the content of saturated, medium-chain FAs, and (2) increase the content of FA with 18 carbon atoms, especially monounsaturated FAs [8]. Aldditionally, free oils are generally not included in cow diets due to the inhibitory effects on the microbial activity in the rumen [43].

Some research has shown that some oils and seeds have better results than others. For example, Roca et al. [29] observed higher concentrations of monounsaturated, polyunsaturated FA, and CLA with the inclusion of cottonseed than with flaxseed. For their part, Kelly et al. [54] obtained higher CLA concentrations in milk with sunflower oil than with peanut and flaxseed, and Kesek et al. [7] obtained better CLA contents with soybean oil than with flaxseed (Table 2).

Oils and seeds with a high content of linoleic acid increase the content of linoleic acid in milk fat; however, the effect on the content of α-linolenic acid is negative with the oils and slightly positive with the seeds [8].

Regarding this, He et al. [59] mentioned that the amounts of vaccenic acid and CLA that flow through the duodenum are greater in cows supplemented with oils rich in linoleic acid compared to cows fed with oils rich in α-linolenic acid. It is worth mentioning that the excess of grains (non-structural carbohydrates), the inclusion of vegetable and marine oils, and the imbalance in the amount of forage in the diet can significantly reduce the amount of fat in the milk [35,63].

### 5.3. Effect of Animal Products on CLA Content in Milk

Cows with high milk production require a high-energy and protein diet; thus, animal products are an option to increase both nutrients in feed mixes [64].

Marine oils (fish, mammals, plankton, or algae) are rich in long-chain polyunsaturated FA, and of these, eicosapentaenoic acid (EPA) and docosahexaenoic acid (DHA) are the most important [65]. These FAs can increase the content of polyunsaturated FAs and CLA in milk. Additionally, they improve reproductive performance and can improve the nutritional value of milk fat in the human diet [66].

Fish oil stimulates the production of vaccenic acid and CLA from linoleic and α-linolenic acids provided by other ingredients that are incorporated into the diet [67]. Despite the benefits of fish oil, it has been shown to decrease fat secretion, as well as that of almost all FAs in milk [65]. Moreover, Juchem et al. [66], observed decreases in true proteins and non-fat milk solids.

This reaction occurs because fish oil causes incomplete biohydrogenation, as it alters the microbial ecosystem [67], and there is an increase in the vaccenic acid flow towards the mammary gland, saturating the activity of the stearyl CoA desaturase enzyme [18].

Table 3 shows the contents of linoleic acid, α-linolenic acid, and CLA from some research work where fish oil was used as an ingredient in the diets of dairy cows.

### 5.4. Effect of Grass Consumption (Grasses and Legumes) on CLA Concentration in Milk

In most production systems, pasture lipids are the main source of fat in the diet of cows [75]. CLA contents in milk from ruminants only grass-fed are higher than in milk from those mixed diets fed, supplements, or concentrates [76]. This increase in CLA in milk fat is because grasses contain higher concentrations of palmitic acid, linoleic acid, and α-linolenic acid [4,14,75] (Table 4).

Linoleic and α-linolenic acids are the precursors of CLA in the process of biohydrogenation and de novo synthesis, respectively [4].

In this regard, Morales et al. [76] mentioned that the fat portion of linoleic and α-linolenic fatty acids is 95%, and of this, between 50 and 75% belongs to α-linolenic acid. The content and composition of FAs are affected by numerous factors, such as: the species and variety of plants, climate, light intensity, rainfall, fertilization, growth stage [77], soil fertility [78], grazing time [79], among others.

About the species, Kalac and Samkova [77] mentioned that milk from cows fresh grass-fed, especially forage legumes, contain more unsaturated FAs and nutritionally beneficial FAs, such as CLA and vaccenic acid, than milk from cows fed with silage or hay (Table 5).

Botana et al. [80] said that forage legumes improve the quality of milk from the point of view of human health. However, Morales [79] states that the concentration of α-linolenic acid is much higher in grasses than in legumes since the lipids of the grasses are found in the leaf chloroplasts; therefore, high consumption of leaves (grasses contain more vegetative material than legumes) could lead to higher consumption of α-linolenic acid and therefore, increase the CLA in milk. Regarding soil fertility, Granados et al. [78] determined that milk from river valley-type soils contains a higher concentration of unsaturated FA, highlighting CLA, compared to savannah-type soils.

In the case of climate, there is a higher content of α-linolenic acid in grasses from temperate zones than in grasses from tropical zones. Moreover, tropical species have a higher total FA content in the dry season than in the rainy season [81]. High temperatures cause a decrease in the content of α-linolenic acid and an increase in palmitic and linoleic acids in plants, especially in grasses. This is due to an adaptation mechanism that decreases the fluidity of the membranes in plant cells to reduce evapotranspiration in high-temperature environments [82].

**Table 4:** Linoleic and α-linolenic acid (g/100 g of fat) contents in some grasses.

Type	Linoleic C18:2	Linolenic C18:3	Authors
Ryegrass (*Lolium perenne*)	15.1	49.8	Aguilar et al. [13]
Kikuyo (*Pennisetum clandestinum*)	10.8	59.5	
Ryegrass (*Lolium perenne*)	12.3	4.5	León et al. [10]
Kikuyo (*Pennisetum clandestinum*)	21.1	30.0	
Chontalpo (*Brachiaria decumbens*)	14.4	20.3	
Estrella (*Cynodon nlemfuensis*)	21.8	27.9	
Alfalfa (*Medicago sativa*)	21.1	30.0	
Alfalfa (*Medicago sativa*)	17.7	30.74	Toyes et al. [82]
Huizache (*Vachellia farnesiana*)	19.03	21.32	
Mezquite (*Vachellia farnesiana*)	8.45	24.44	
Palo fierro (*Olneya tesota*)	11.85	31.34	
Palo verde (*Parkinsonia aculeata* L.)	13.33	37.34	
Amargo (*Paspalum conjugatum*)	0.40	0.46	Mojica et al. [12]
Llanero (*Andropogon gayanus*)	0.33	0.37	
Estrella (*Cynodon nlemfuensis*)	0.39	0.11	
Humidícola (*Brachiaria humidicola*)	0.32	0.12	
Mombaza (*Panicum maximum*)	0.69	0.47	
Elefante (*Pennisetum purpureum*)	0.23	0.35	
Mulato (*Brachiaria ruziziensis*)	0.73	0.54	Mojica et al. [81]
Tanzania (*Panicum maximum* cv.)	0.80	1.20	
Toledo (*Brachiaria brizantha*)	0.99	1.08	

**Table 5:** Effects of different feeding systems (grass, silage, hay, TMR) on linoleic, α-linolenic, and CLA (g/100 g of fat) contents in cow’s milk.

Feeding Systems	Linoleic C18:2	Linolenic C18:3	CLA C18:2 Cis-9 Trans-11	Author
**Grass**				
Grasses	0.67	0.53	1.55	Mojica et al. [81]
Legumes	0.70	0.17	2.24	Aguilar et al. [13]
**Silage**				
Alfalfa (Medicago sativa)	4.51	1.11	1.30	Castro et al. [62]
Corn	1.77	0.12	0.88	Barletta et al. [83]
**Hay**				
Alfalfa (Medicago sativa)	2.70	0.91	1.80	Aprianita et al. [84]
Oat	0.87	0.81	0.87	Caroprese et al. [85]
**TMR**				
Corn silage	0.34	0.23	0.78	Lucia et al. [86]
Sorghum silage	2.37	0.49	0.85	Rego et al. [87]

TMR; total mixed ration.

Grazing intensity also influences the lipid profile of milk and especially of CLA precursors. Mojica et al. [81] recorded higher contributions of linoleic acid and α-linolenic acids with high-intensity grazing. Likewise, Morales et al. [79] argued that grazing for 12 h provides higher contents of α-linolenic acid and CLA in milk, given the higher intake of green matter.

The increases in CLA are not necessarily attributed to the increase in α-linolenic acid in the diet but rather to an effect on ruminal fermentation. The presence of secondary compounds in plants, such as polyphenols and terpenoids, can inhibit the microorganisms that hydrogenate in the rumen. Tannins have been shown to affect the decrease of Butyrivibrio fibrisolvens strains, one of the most important species in the biohydrogenation process [10]. This inhibition results in an increase in the flow of vaccenic acid from the rumen to the mammary gland [81].

Feeding ruminants with higher amounts of polyunsaturated FAs can inhibit the last stage of the biohydrogenation process in the rumen; thus, there would be a greater amount of FA precursors of CLA and a greater flow of these towards the small intestine where they are absorbed and transported to the mammary gland before being excreted in the milk [9]. In addition, fresh grass increases rumen pH and affects the species of microorganisms that induce biohydrogenation, with CLA production being higher at pH greater than 6.0 [4].

## 6. Effect of Agronomic Management of Pastures to Increase the Amount of CLA Precursors

There are two possible ways to increase the concentration of FAs in milk; one of them is by increasing the substrate concentration in the feed, and another is by reducing the biohydrogenation degree in the rumen [88]. The FAs with the highest proportion in fresh grass are: α-linolenic, palmitic, and linoleic; the first and the third are the precursors of CLA [13]. The total concentration of long-chain FAs (linoleic and α-linolenic) will increase with management techniques that promote rapid vegetative growth [11]. Among these techniques are the management of regrowth age and nitrogen fertilization [12].

A positive correlation between nitrogen concentration and FAs has been found. León et al. [10] stated that the high availability of nitrogen stimulates the production of dry matter, increases the number of leaves, and stimulates the synthesis of metabolic components, including chlorophyll and leaf protein. Nitrogen fertilization could cause greater synthesis and accumulation of lipids in plants [89], as well as a greater amount of FAs since these are located within the cellular content of the plant [10].

Elgersma et al. [88] recorded significant increases in palmitic (18%), linoleic (12%), and α-linolenic (40%) acids in the grass and a general increase of 26% in the concentration of total FA when 120 kg nitrogen per hectare was applied. Boufaïed et al. [89] stated that the effects of nitrogen fertilization depend on the growth stage of the grass since they observed decreases in oleic and α-linolenic acids at an earlier growth stage when the grass was not fertilized.

Respecting the regrowth age, Mojica et al. [60] stated that the reduction in the concentration of FA is associated with a lower leaf–stem ratio in mature forage since FAs are especially concentrated in chloroplasts, and the reduction of this component in the plant negatively affects the content of FA. Aguilar et al. [13] mentioned that with young forages a higher concentration of CLA in milk is expected than with mature forages because there is a higher content of α-linolenic acid, and the fiber level is lower than with increasing age of the forage. In addition to the effect of the leaf–stem ratio, other factors, such as leaf maturity, flowering initiation, and senescence also negatively affect the concentration of FAs in the forage [12]. Elgersma et al. [88] stated that an increase in the regrowth period from 20 to 38 days produced a very significant decrease in the level of total FAs and found lower concentrations of α-linolenic and palmitoleic acids and higher content of stearic and linoleic acids after a longer regrowth period.

Likewise, Mojica et al. [12] observed that in forage with three weeks of regrowth, the content of linoleic acid was higher than that of α-linolenic acid, but the content of the latter in relation to linoleic acid tended to be higher in the sixth and ninth weeks of regrowth. It is in this way that nitrogen fertilization in sync with the management of short regrowth periods could increase the content of total FAs in pastures and especially that of CLA precursors.

## 7. Conclusions

The amount of CLA in milk is produced by the biohydrogenation process in the rumen and by endogenous synthesis (de novo) in the mammary gland, while the chemical composition of milk and the acid profile are modified by intrinsic factors and extrinsic. Of the extrinsic factors with the greatest influence on the amount of fat in milk and the profile of fatty acids is the diet.

The content of CLA in the milk of cows will depend on the chemical composition and fatty acid profile, especially the concentrations of linoleic and α-linolenic acids of the forages used in animal diets, as well as the interaction of these with the microbial flora of the rumen, responsible for the biohydrogenation process. The concentrations of linoleic and α-linolenic acids in forages are affected by factors, such as the nature of the forage and how they are supplied or the treatments to which they are subjected.

With the inclusion of oilseeds, vegetable oils, and agricultural byproducts, better results can be obtained in the CLA content in milk due to the large amount of polyunsaturated FAs they contain. However, excessive use of these products could cause a decrease in fat percentage. The addition of fresh grasses in the diet of cows has helped to improve the CLA content in milk. With nitrogen fertilization and regrowth age management, pastures improve their lipid profile and provide greater amounts of linoleic and α-linolenic acids to the diets of cows to increase the amount of CLA in milk and obtain food of a higher nutritional quality.

## Figures and Tables

**Table 1:** Contents of linoleic C18:2, linolenic C18:3, and CLA FA in milk (g/100 g of fat) using different types of seeds in the feeding of dairy cows.

Type	Inclusion (% DM of Diet)	Linoleic C18:2	Linolenic C18:3	CLA C18:2 Cis-9 Trans-11	Authors
**Seeds**					
Soybean (extruded)	11.97	5.62	0.96	00.69	Dhiman et al. [43]
Cotton (extruded)	11.97	4.38	0.50	0.60	
Flax	41	2.25	1.21	1.16	Ward et al. [44]
Rapeseed	41	2.16	0.48	1.41	
Canola	14	1.72	0.52	0.39	Cichlowski et al. [45]
Flax (raw)	12.5	2.7	1.3	1.4	Gonthier et al. [46]
Flax (micronized)	12.7	2.9	1.3	1.4	
Flax (extruded)	12.7	3.1	1.0	1.9	
Rapeseed (ground)	41	1.99	0.56	0.68	Egger et al. [47]
Flaxseed (extruded)	46	2.25	1.18	0.8	
Sunflower	11.2	++	++	2.05	Silva et al. [48]
Soybean (toasted)	7.5	3.46	0.34	8.85	Liu et al. [49]
Flax (toasted)	7.5	3.05	0.41	8.82	
Sunflower (toasted)	7.5	2.96	0.27	0.72	
Peanut (toasted)	7.5	3.03	0.27	0.66	
Cotton (toasted)	7.5	2.76	0.29	0.63	
Flax (raw)	12.4	2.05	0.65	0.98	Chilliard et al. [50]
Flax (extruded)	21.2	4.21	1.2	1.33	
Flax	14	++	++	1.03	Fuentes et al. [51]
Cotton	12	++	++	0.99	
Cotton	12	2.98	0.51	1.04	Roca et al. [29]
Flax	20	2.81	0.57	0.91	

++ No data logging: DM; dry matter.

**Table 2:** Contents of linoleic C18:2, linolenic C18:3, and CLA FA in milk (g/100 g of fat) using different types of oils in the feeding of dairy cows.

Type	Inclusion (% DM of Diet)	Linoleic C18:2	Linolenic C18:3	CLA C18:2 Cis-9 Trans-11	Authors
**Oils**					
Peanut	5.3	2.36	0.18	1.33	Kelly et al. [54]
Sunflower	5.3	2.78	0.19	2.44	
Flax	5.3	3.27	0.44	1.67	
Cotton	2	3.43	++	0.60	Zheng et al. [55]
Soybean	2	3.87	++	1.02	
Corn	2	2.95	++	0.69	
Rapeseed	1.5	3.48	0.42	0.97	Brzóska [56]
Flaxseed	1.5	3.16	0.35	0.81	
Soybean	1.5	3.28	0.34	0.90	
Sunflower	1.5	3.45	0.40	1.05	
Flaxseed	5	3.50	0.95	2.02	Roy et al. [57]
Sunflower	5.2	2.62	0.15	2.18	
Sunflower	5.1	4.29	0.12	1.94	
Flaxseed	2.57	2.62	0.78	1.18	Flowers et al. [58]
Flaxseed	5.12	2.95	1.01	1.39	
Flaxseed	7.67	3.33	1.03	1.65	
Palm oil	5	2.22	0.53	1.51	He y Armentano [59]
Flaxseed	5	2.57	0.82	2.67	
Corn	5	2.99	0.55	3.68	
Safflower	5	3.34	0.56	4.09	
Sunflower	2	3.68	0.44	1.92	Prieto et al. [42]
Sunflower	4	4.07	0.32	2.24	
Canola	3	1.34	0.36	0.52	Welter et al. [60]
Canola	6	1.33	0.35	0.60	
Soybean	3	++	++	1.49	Vieyra et al. [61]
Soybean	6	++	++	1.40	
Soybean	2.3	5.01	0.82	0.65	Castro et al. [62]
Flax	2.3	4.51	1.30	1.11	

++ No data logging: DM; dry matter.

**Table 3:** Contents of linoleic acid, α-linolenic acid, and CLA in cow’s milk (g/100 g of fat) using fish oil.

Inclusion (% DM of Diet)	Linoleic C18:2	Linolenic C18:3	CLA C18:2 Cis-9 Trans-11	Author
3	2.64	0.55	1.90	Donovan et al. [68]
2	2.99	0.57	2.43	Baer et al. [69]
2	2.20	0.85	0.88	Abughazahiet et al. [67]
0.67	3.15	0.81	1.19	Whitlock et al. [70]
3.5	1.4	0.64	2.16	Murphy et al. [71]
1	2.47	0.34	1.66	Toral et al. [72]
2	1.88	0.71	2.45	Kupczynski et al. [73]
2	1.27	0.30	1.81	Alizadeh et al. [74]

DM; dry matter.

## Data Availability

Not applicable.
